# The Intestinal and Biliary Metabolites of Ibuprofen in the Rat with Experimental Hyperglycemia

**DOI:** 10.3390/molecules27134000

**Published:** 2022-06-22

**Authors:** Hawsar Othman Mohammed, Attila Almási, Szilárd Molnár, Pál Perjési

**Affiliations:** 1 Institute of Pharmaceutical Chemistry, Faculty of Pharmacy, University of Pécs, H-7624 Pécs, Hungary; hawsar.mohamed@univsul.edu.iq (H.O.M.); attila.almasi@aok.pte.hu (A.A.); molnar.szilard@freemail.hu (S.M.); 2 College of Veterinary Medicine, University of Sulaimani, Sulaymaniyah 46001, Iraq

**Keywords:** ibuprofen, streptozotocin, hyperglycemia, intestinal metabolism, hepatic metabolism, HPLC-MS

## Abstract

Hyperglycemia is reported to be associated with oxidative stress. It can result in changes in the activities of drug-metabolizing enzymes and membrane-integrated transporters, which can modify the fate of drugs and other xenobiotics; furthermore, it can result in the formation of non-enzyme catalyzed oxidative metabolites. The present work aimed to investigate how experimental hyperglycemia affects the intestinal and biliary appearance of the oxidative and Phase II metabolites of ibuprofen in rats. In vivo studies were performed by luminal perfusion of 250 μM racemic ibuprofen solution in control and streptozotocin-treated (hyperglycemic) rats. Analysis of the collected intestinal perfusate and bile samples was performed by HPLC-UV and HPLC-MS. No oxidative metabolites could be detected in the perfusate samples. The biliary appearance of ibuprofen, 2-hydroxyibuprofen, ibuprofen glucuronide, hydroxylated ibuprofen glucuronide, and ibuprofen taurate was depressed in the hyperglycemic animals. However, no specific non-enzymatic (hydroxyl radical initiated) hydroxylation product could be detected. Instead, the depression of biliary excretion of ibuprofen and ibuprofen metabolites turned out to be the indicative marker of hyperglycemia. The observed changes impact the pharmacokinetics of drugs administered in hyperglycemic individuals.

## 1. Introduction

**Figure 1:**
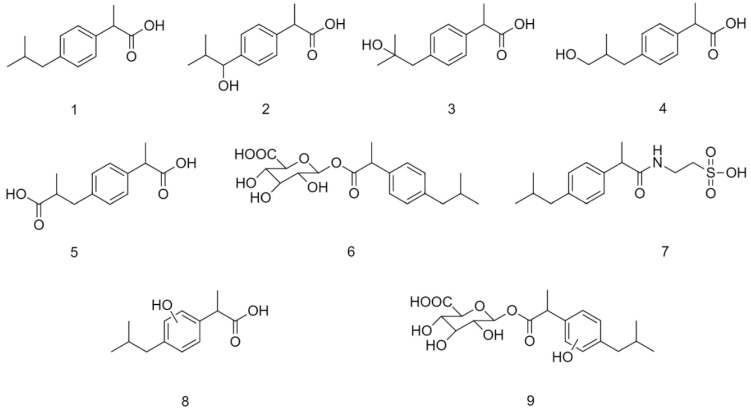
Structural formula of plausible metabolites of ibuprofen (IBP) (**1**): 1-hydroxyibuprofen (1-OH-IBP) (**2**), 2-hydroxyibuprofen (2-OH-IBP) (**3**), 3-hydroxyibuprofen (3-OH-IBP) (**4**), carboxyibuprofen (HOOC-IBP) (**5**), ibuprofen-β-D-glucuronide (IBP-GLU) (**6**), ibuprofen taurate (IBP-TAU) (**7**), hydroxylated ibuprofen (**8**), and glucuronide conjugate of a hydroxylated ibuprofen (**9**). (The position of the hydroxyl substitution of **8** and **9** is uncertain).

Ibuprofen (IBP) (**1**) is a non-steroidal anti-inflammatory drug (NSAID) belonging to the group of 2-arylpropionic acids (Figure 1). It is an alkylbenzene with a carboxylic acid functional group [1]. The molecule, carrying a center of symmetry, is used in therapy in racemic form. It is most commonly used by oral administration. Absorption is mediated mainly from the small intestine; however, the role of the stomach is not negligible either. After absorption, the (*R*)-enantiomer of the racemic mixture undergoes unidirectional (*R*)–(*S*) transformation in the liver resulting in formation of the pharmacologically more active enantiomer [2].

Over 70% of ibuprofen is metabolized and excreted in the urine. The major route of metabolism of the parent compound is oxidative conversion catalyzed by CYP enzymes, resulting in the formation of hydroxyl-substituted ibuprofen metabolites, 1-hydroxy-(1-OH-IBP) (**2**), 2-hydroxy-(2-OH-IBP) (**3**), and 3-hydroxyibuprofen (3-OH-IBP) (**4**). The latter is oxidized to the corresponding 3-carboxy derivative (HOOC-IBP) (**5**) in the cytosol (Figure 1). Both IBP and its oxidized metabolites were reported to form glucuronic acid conjugates (**6**) excreted in the feces or the urine [1,2,3].

Reactive oxygen species (ROS) can be found in the background of most inflammatory (e.g., rheumatoid arthritis) and neurodegenerative diseases (e.g., Alzheimer’s disease, Parkinson’s disease) and in the pathomechanism of cardiovascular diseases and metabolic disorders (e.g., diabetes) [4,5,6,7]. They are capable of oxidizing endogenous and exogenous molecules in non-specific non-enzyme-catalyzed reactions. For example, while diabetic individuals were treated with acetylsalicylic acid, a significant increase in the plasma level of salicylate metabolites (e.g., 2,3-dihydroxybenzoic acid) produced exclusively by a non-enzyme-catalyzed hydroxylation reaction has been observed [4].

Diabetes mellitus is a complex endocrine metabolic disorder that affects a large proportion of the world’s population. It is a well-known risk factor for cardiovascular disease and atherosclerotic complications, especially coronary heart disease. Hyperglycemia is a leading factor in diabetic complications, inducing tissue damage through different pathways [7]. These biochemical pathways generate reactive oxygen species (ROS), increasing oxidative stress [7,8]. One of the major sources of ROS formation in diabetic patients is the vascular type (Nox) NADPH oxidases [9]. Nox enzymes are, however, not the only source of ROS under hyperglycemic conditions; mitochondria and uncoupling of endothelial NO synthase also contribute to oxidative stress [10].

Strong research evidence has been accumulated to indicate that disease–drug interactions can profoundly affect the response to medication [11]. Changes in the amount and function of enzymes and transporters may alter the pharmacokinetics of the compounds [11,12,13]. Earlier, we investigated how the activity of the synthesizing and hydrolytic enzymes involved in glucuronide and sulfate conjugation were affected in streptozotocin (STZ)-treated rats. Activity of the UDP-glucuronyltransferase was significantly decreased in the liver of the STZ-treated rats. On the other hand, activity of the sulfotransferase and the respective hydrolytic enzymes did not change significantly [14]. According to these observations, we found the biliary excretion of ibuprofen glucuronide (IBP-GLU) in the STZ-treated (hyperglycemic) experimental animals statistically lower than in the controls [15].

As a continuation of these earlier studies, in our present experiment, biliary excretion of the oxidative and Phase II metabolites of IBP was investigated in the control and the STZ-treated rats. By application of the previously used in vivo protocol [14,15,16], we investigated how hyperglycemic conditions affect the intestinal and biliary appearance of the oxidative and Phase II metabolites of IBP. The biliary oxidative profiles were compared with those of two in vitro tests (Fenton and Udenfriend tests), in which reactive oxygen species (hydroxyl radicals) are supposed to react with IBP in non-enzyme-catalyzed reactions [17].

## 2. Results

### 2.1. Blood Glucose Level

Diabetes was induced with a single iv dose of 65 mg/kg STZ [18]. Hyperglycemia was confirmed after one week of the STZ-treatment [19]. The average blood glucose level of the control animals was 6.7 ± 1.5 mM, while that of the STZ-treated rats was 23.4 ± 2.8 mM.

### 2.2. Fenton Tests

Earlier, we investigated how the ratio of the molar concentration of the substrate (salicylic acid) to the iron(II) ion in the Fenton test affects the effectiveness of the hydroxylation reaction. The present investigations were performed using the more effective conditions, using the substrate: iron(II): hydrogen peroxide molar ratio as 1:3:1 [20].

**Figure 2:**
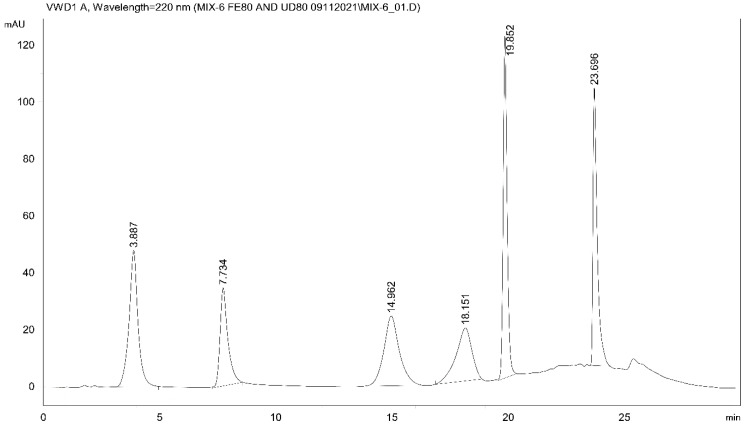
HPLC-UV chromatogram (Method I) of IBP and oxidative metabolites of IBP in ACN (30 μg mL^−1^ each). The retention times (t_R_) of the separated standards are as follows: 3-OH-IBP (**4**) (3.89 min), SA (internal standard) (7.73 min), 2-OH-IBP (**3**) (14.96 min), HOOC-IBP (**5**) (18.15 min), 1-OH-IBP (**2**) (19.85 min), and IBP (**1**) (23.70 min).

For analysis of the samples, a high-performance liquid chromatographic gradient method (Method I) with UV detection (HPLC-UV) was developed to separate the available oxidative metabolites of IBP (Figure 2).

HPLC-UV analysis (Method I) of the Fenton extracts indicated the formation of 1-OH-IBP (**2**), 2-OH-IBP (**3**), and several other products (Figures S1 and S2). To verify the structures of **2** and **3** and to identify other oxidation products, a high-performance liquid chromatographic analysis using mass spectrometric detection (HPLC-MS) of the extracts was performed. HPLC-MS investigations confirmed the presence of **2** (*m*/*z* 221.1178), **3** (*m*/*z* 221.1178), and HOOC-IBP (**5**) (*m*/*z* 235.0969) (Figures S3–S5). The derivative (**8**) with the highest HPLC-UV integrated peak area (t_R_ = 22.05 min) could not be unambiguously identified (Figure 1). The formed product (X-HO-IBP) has an exact mass of *m*/*z* 221.1175 and its fragmentation makes no distinction between the 2′-OH-IBP and OH(Ar)-IBP structures (Figure S6A,B). Another IBP-derived HPLC-UV peak (t_R_ = 21.09 min) was identified as a dihydroxy ibuprofen (**10**) (OH-IBP-OH) derivative (*m*/*z* 237.1125) (Figure S7). Determination of the exact structures needs further investigation. The change in HPLC-UV integrated peak areas of **2**, **3**, **8**, and **10** (relative to that of the internal standard) as a function of incubation times is shown in Figure S8.

### 2.3. Udenfriend’s Test Results

Using the in vitro non-enzyme-catalyzed hydroxylation test developed by Udenfriend et al., [21,22], a similar oxidative metabolic pattern of IBP could be observed. Similar to the Fenton incubations, the derivatives with the t_R_ values of 22.03 min (**8**) and 21.07 min (**10**) were those with the highest HPLC-UV peak areas (Figures S9 and S10). 2-HO-IBP (**3**) was formed in lower amounts than in the respective Fenton samples (Figure S11). 1-OH-IBU (**2**) could not be detected. HPLC-MS investigations of the samples confirmed the presence of the **2**, **3**, **5**, **8**, and **9** derivatives.

### 2.4. Analysis of Intestinal Perfusate and Bile Samples

For HPLC-UV analysis of the biological samples, a modified method was developed (Method II) to separate the oxidative metabolites and the glucuronide conjugate of IBP (Figure S12). HPLC-UV analysis of the ether extract of the intestinal perfusate samples of the control and the hyperglycemic animals did not indicate either hydroxy- or carboxyibuprofen metabolites (Figures S13–S15). However, contrary to our previous HPLC-UV measurements [15], IBP-GLU (**6**) (*m*/*z* 381.1552) could be identified by HPLC-MS in the perfusate of both the control and the STZ-treated animals (Figure S16).

**Figure 3:**
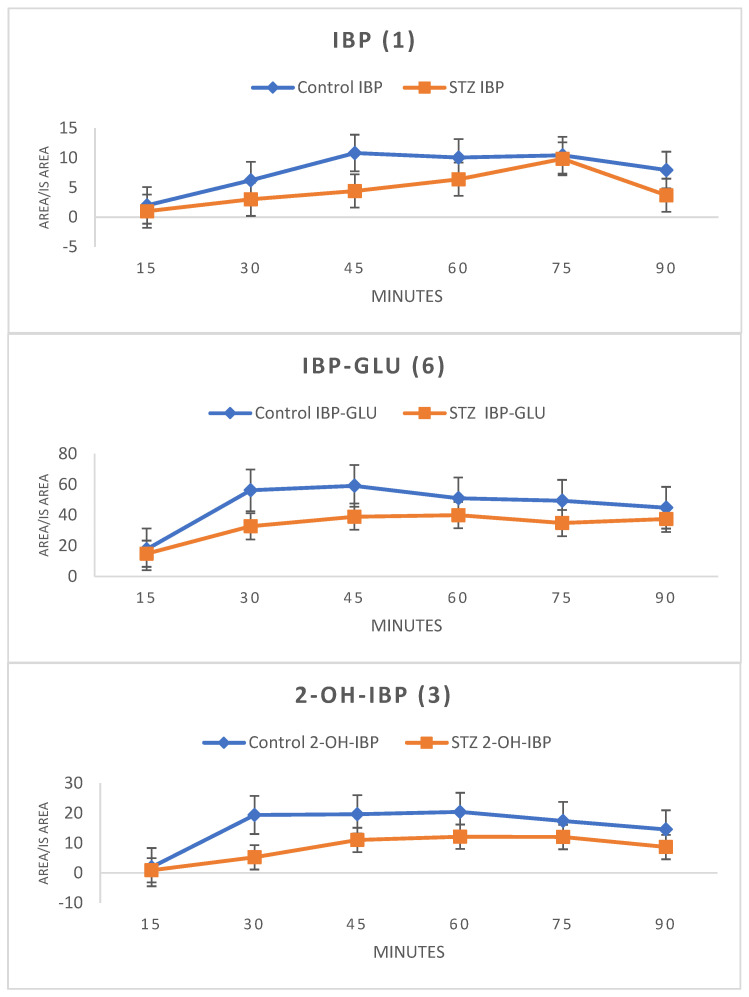
Change in the HPLC-UV integrated peak areas (relative to the internal standard) of ibuprofen (**1**) and the ibuprofen metabolites (IBP-GLU (**6**) and 2-OH-IBP (**3**)) in the diethyl ether extract of bile of control and hyperglycemic (STZ) rats (Method II).

HPLC-UV analysis of the ether extract of bile samples showed the presence of IBP (**1**), IBP-GLU (**6**), and 2-OH-IBP (**3**). HPLC-MS analysis confirmed the presence of **1** (*m*/*z* 205.1229), **6** (*m*/*z* 381.1549), and **3** (*m*/*z* 221.1178) in both the control and the hyperglycemic samples. To keep the number of experimental animals low, the results were evaluated by comparing the integrated HPLC-UV peak areas (relative to the internal standard) of the compounds. The areas were lower in the bile samples of the STZ-treated animals at each investigated timepoint (Figure 3).

**Figure 4:**
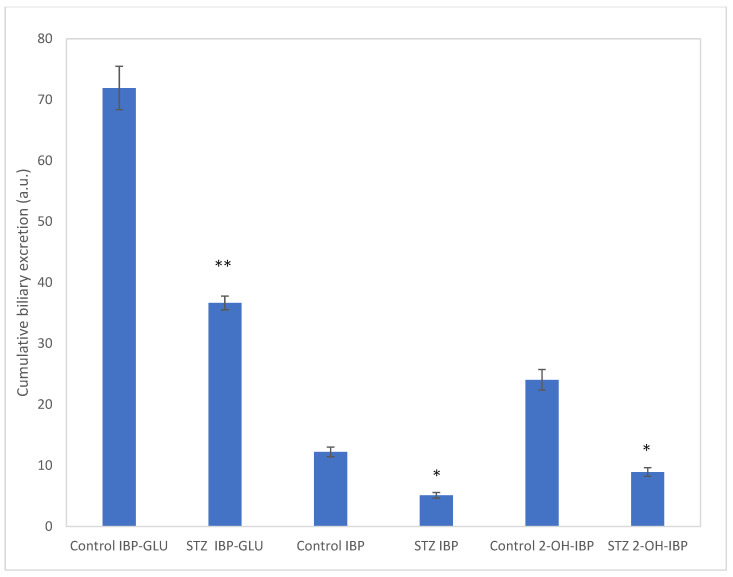
Cumulative biliary excretion (HPLC-UV (Method II) based integration) of IBP (**1**), IBP-GLU (**6**), and 2-OH-IBP (**3**) after the 90-min luminal perfusion of 250 μM IBP in control and hyperglycemic (STZ-treated) rats. Each value represents the average of five independent experiments ± standard error. Significant difference from the control value: * *p* < 0.05, ** *p* < 0.01.

Considering the respective bile outflows, the relative amounts of the cumulative excretions of **1**, **6**, and **3** were calculated. The result showed a depression in biliary excretion of all the three compounds in the STZ-treated animals. In our earlier experiments (using the same experimental protocol), the cumulative biliary excretion of IBP was reduced by 53% [15]. The present data indicated similar (58%) depression. For IBP-GLU and 2-OH-IBP, the excretion was depressed by 63% and 49%, respectively (Figure 4). Additionally, several other peaks appeared in the extracts (Figures S17–S19). Based on the HPLC-MS analysis of the samples, these peaks are bile acids and conjugated bile acid derivatives. Based on their high-resolution mass spectra, cholic acid (exact mass: 407.2798), glycocholic acid (exact mass: 464.3012), taurochenodeoxycholic acid (exact mass: 498.2889), and taurocholic acid (exact mass: 514.2839) was identified.

**Figure 5:**
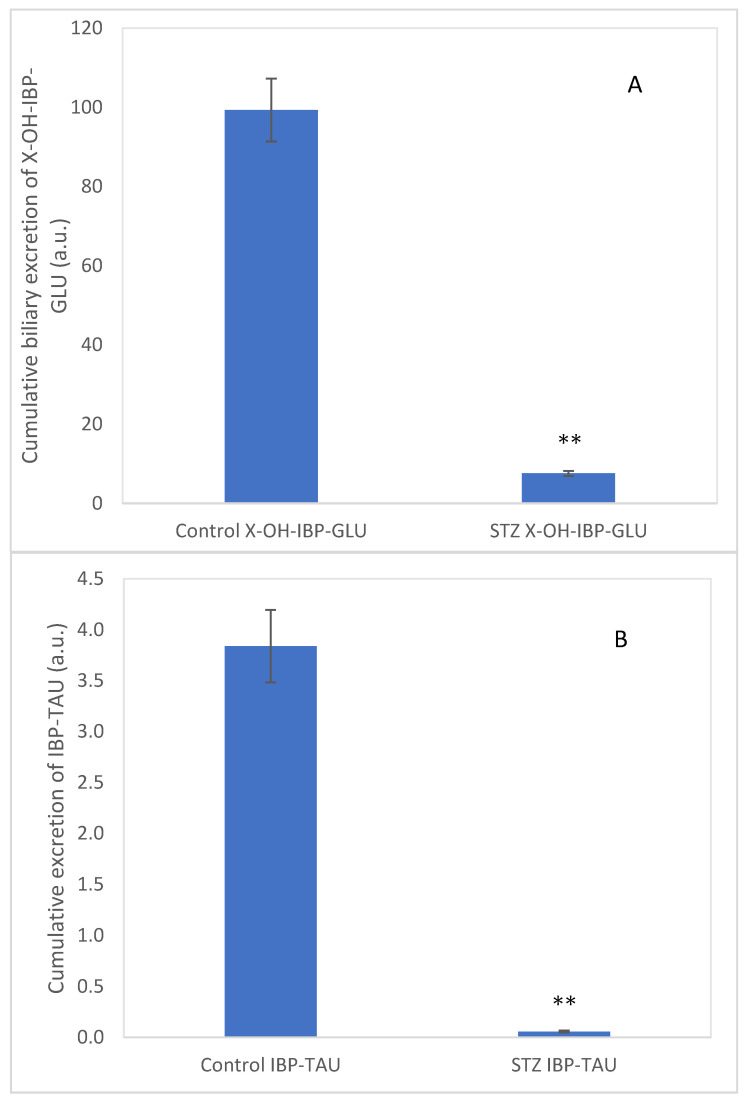
Cumulative biliary excretion (HPLC-MS based integration) of the (**A**) X-OH-IBP-GLU (**9**) and (**B**) IBP-TAU (**7**) after the 90-min luminal perfusion of 250 μM IBP in control and hyperglycemic (STZ) rats. Each value represents the average of five independent experiments ± standard error. Significant difference from the control value: ** *p* < 0.01.

Furthermore, an HPLC-MS analysis of the bile samples confirmed the presence of the glucuronide conjugate of a hydroxylated ibuprofen (**9**) (exact mass: 397.1501) (X-OH-IBP-GLU) and the taurine conjugate of ibuprofen (**7**) (exact mass: 312.1275) (IBP-TAU) (see Figures S20 and S21, respectively). The cumulative biliary excretion of X-OH-IBP-GLU and IBP-TAU in the control and the STZ-treated rats are shown in Figure 5A,B. The excreted X-OH-IBP-GLU and IBP-TAU were depressed by 92% and 98%, respectively.

## 3. Discussion

Ibuprofen (IBP) undergoes extensive Phase I and Phase II biotransformation to the main metabolites 1-OH-IBP (**2**), 2-OH-IBP (**3**), HOOC-IBP (**5**), and IBU-GLU (**6**) [1,2,3]. In humans, the CYP2C9 isoform plays the most important role in the oxidative metabolism of IBP, mediating the 2- and 3-hydroxylations and the subsequent 3-oxidation in the liver [23,24]. Additional CYPs, particularly CYP2C8, may also play a role in these biotransformations [24,25]. The enzyme responsible for the 1-OH-IBP (**2**) formation has not been identified [26,27].

In rats, the most abundant CYP2C isoform is CYP2C11, whereas the other isoforms were present at much lower levels. The human CYP2C9 and CYP2C8 proteins are orthologous to the rat CYP2C12 and CYP2C11 [28]. After oral administration, IBP appeared mainly in an unchanged form in the plasma of rats. In addition, two metabolites, 2-OH-IBP (**3**) and HOOC-IBP (**5**), were found in the plasma and the urine [29]. Yamazoe et al., studied the effect of alloxan and STZ on the expression of hepatic CYP enzymes in rats. In most cases, the expression of the CYP proteins was either not affected or depressed [30]. Later, Shimojo reviewed the effects of hyperglycemia on rat hepatic CYP expressions. According to the reviewed publications, the amount of CYP2C11 and CYP2C13 was suppressed, while CYP2C12 did not change or somewhat increased [31]. Recently, Yang and Liu reviewed the effect of diabetes on the drug transporter–CYP interplays. They referred to further studies that demonstrate a lowered expression of hepatic CYP2C11 in diabetic rats [13]. Accordingly, decreased levels of hydroxylated IBP metabolites formed in CYP-catalyzed reactions can be rationalized.

During inflammation, for example, stimulated polymorphonuclear leukocytes (PMN) and macrophages produce large amounts of superoxide ions and hydrogen peroxide [32]. There are many examples of exogenous H_2_O_2_ initiating redox signals or stress responses. Furthermore, receptor-mediated redox signaling is widely regarded as involving endogenously generated H_2_O_2_ [32,33]. On the other hand, many of the biologically damaging effects of H_2_O_2_ depend on transition metals such as iron and copper, which cleave the peroxide bond to generate hydroxyl radicals or activated metal complexes [34]. Hydroxyl radicals are powerful electrophilic reactants that can oxidize endogenous or exogenous molecules. Therefore, the importance of non-enzymatic oxidation can be particularly significant at the sites of inflammation [35].

To model non-enzymatic oxidation, oxidation products of IBP formed under the conditions of the Fenton reaction [36] and Udenfriend’s oxidation [21,22] were investigated by HPLC-UV and HPLC-MS methods. The Fenton reaction is a tool for modeling oxidative stress by forming hydroxyl radicals in a reaction of hydrogen peroxide and some transitional metals. The key features of the reaction are believed to be the reaction conditions, such as reagent concentrations, pH, and temperature [37]. Earlier, we applied the reactions to study the oxidative transformation of salicylates [20,38].

The Fenton oxidation of IBP resulted in the formation of several products. Among them, 1-OH-IBP (**2**), 2-OH-IBP (**3**), and HOOC-IBP (**5**) were identified by HPLC-UV and HPLC-MS. Among these products, 1-OH-IBP (**2**) was found in the largest amount (Figure 1). Additionally, a small amount of a dihydroxyibuprofen (t_R_ = 8.91 min) (**10**) and a significant amount of a new hydroxyl-substituted derivative (t_R_ = 9.24 min) (**8**) were found. Hydroxyl radicals have been reported to react with IBP to form 1-OH-(**2**), 2′-OH-, and OH(Ar)-IBP derivatives [39]. Based on the HPLC retention time (t_R_= 9.24 min), the new hydroxyl derivative could be the 2′-OH-IBP or an OH(Ar)-IBP. As shown in Figure S6B, the main fragment ion (C_12_H_17_O; *m*/*z* 177.1251) is formed by the CO_2_ loss of the hydroxylated IBP derivative (*m*/*z* 221.1175). The CO_2_ loss occurs already in the ionization source (Figure S6A). The other main decomposition peak (*m*/*z* 119.0472) corresponds to a C_8_H_7_O (vinyl phenol or acetophenone; exact mass: *m*/*z* 119.0497, mass error: −21.0 ppm) fragment [40]. Although the *m*/*z* 149.0576 peak can be assigned as a C_9_H_9_O_2_ fragment (exact mass *m*/*z* 149.0603; mass error: −18.1 ppm), the exact structure of the formed product cannot be unambiguously determined. Since H-abstraction is thermodynamically more favored than the OH addition and its reaction rate is an order of magnitude higher [41,42], the new hydroxylated product was tentatively identified as the 2′-OH-IBP (Figure S6A,B). Further investigations are to be performed to experimentally prove the results of the theoretical calculations. 1,2-Dihydroxyibuprofen was reported to form in the biodegradation of IBP [43].

Udenfriend et al., described a system consisting of EDTA, ascorbic acid, molecular oxygen, and iron(II) ion, which would serve as a model of the biomimetic hydroxylation of organic substrates [21,22]. Ito et al., demonstrated that the reactive species are hydroxyl radicals (trapped by different aliphatic alcohols) in both the Fenton and the Udenfriend reagents [44]. Udenfriend’s oxidation of IBP resulted in the formation of products similar to those formed in the Fenton incubations (Figure 4). Based on this observation, it is reasonable to presume that the reactive species are the same in both reactions. The above results indicate that in vivo Fenton-type [37,45] or Udenfriend-type [46] non-enzymatic hydroxylation of IBP—based on measurement of **8** and/or **9**—can be used as an early sign of oxidative stress conditions.

While analyzing the 250 μM ibuprofen containing intestinal perfusates of control and STZ-treated hyperglycemic rats, none of the oxidative metabolites (formed in CYP-catalyzed or non-CYP-catalyzed reactions) of IBP (**1**) could be detected by HPLC-UV or HPLC-MS. Contrary to the previous [15] (and the present) HPLC-UV measurements, IBP-GLU (**6**) conjugate could be detected and identified in the perfusates by HPLC-MS. This result indicates the formation of **6** in the rats’ small intestine, which could not be detected by the less sensitive UV detection.

HPLC-UV analysis of the collected bile samples showed the presence of IBP (**1**), IBP-GLU (**6**), and 2-OH-IBP (**3**). HPLC-MS analysis of the bile samples of both the control and the hyperglycemic group of animals confirmed the presence of the three compounds. According to our previous results [15], excretion of both **1** and **6** into the bile decreased in experimental diabetes; similarly, biliary excretion of **3** was also reduced in the hyperglycemic rats (Figure 4). In our earlier experiments, the biliary excretion of 4-nitrophenol and its glucuronic acid and sulfate conjugates were significantly decreased in diabetic rats [16].

In addition to the above, the glucuronide conjugate of a hydroxylated IBP (**9**) and the taurine conjugate of IBP (**7**) could also be identified by means of HPLC-MS (Figures S20 and S21). Earlier, Shirley et al., reported on the formation of IBP-CoA and IBP-TAU (**7**) in in vitro incubations using rat hepatocytes [47]. Similar to those results, the HPLC-MS analysis of our bile samples indicated the presence of IBP-TAU (**7**) but not of the glycine conjugate (IBP-GLY). To the best of our knowledge, this is the first in vivo observation of the excretion of IBP-TAU into the bile.

The excreted amounts of **9** and **7** were significantly lower in the STZ-treated rats’ bile samples. Although the bile outflow was slightly increased in the STZ-treated animals over the studied period, a comparison of the relative amounts of the excreted **9** and **7** (based on the HPLC-MS data) indicated reduced excretion of IBP-TAU in the STZ-treated animals (Figure 5). Depression of biliary excretion of IBP-TAU can be explained by the earlier results related to the reduced biliary excretion of IBP and IBP-GLU in hyperglycemic rats [48,49,50]. Hasegawa et al., [46] reported that the hepatic expression of the efflux transporter MRP2 is decreased in the STZ-treated rats. Similarly, expression of the efflux transporter P-gp (MDR1) was reduced in hyperglycemia [49,50,51]. In agreement with these earlier results, our molecular biology studies on liver samples of the STZ-treated rats showed decreased expression of the efflux transporters P-gp (MDR1B), MRP2, and BCRP [15]. These transporters are involved in exporting organic anions from the hepatocytes into the bile canaliculus [52]. Furthermore, hyperglycemia is also associated with a decline in the levels of the endogenous antioxidant taurine in several tissues [53,54].

## 4. Materials and Methods

### 4.1. Chemicals

1-Hydroxyibuprofen (1-OH-IBP) and 2-hydroxyibuprofen (2-OH-IBP) were obtained from Dr. Ehrenstorfer GmbH (Augsburg, Germany). Ibuprofen (IBP), salicylic acid (SA), streptozotocin (STZ), ibuprofen-β-D-glucuronide (IBP-GLU), and carboxyibuprofen (HOOC-IBP) were purchased from Sigma-Aldrich (Budapest, Hungary). 3-Hydroxyibuprofen (3-OH-IBP) was from HPC Standards GmbH (Cunnersdorf, Germany). All chemicals and reagents were analytical or HPLC grade. The standard isotonic perfusion medium had the following compositions (mM): NaCl 96.4, KCl 7.0, CaCl_2_ 3.0, MgSO_4_ 1.0, sodium phosphate buffer (pH 7.4) 0.9, tris buffer (pH 7.4) 29.5, glucose 14.0, mannitol 14.0. Blood glucose level was checked with an AccuChek blood glucose meter (Roche).

### 4.2. Fenton Test

The experiments were performed as described before [20]. Iron(II) sulfate (100 µL of 30 mM) solution (in pH 3.0 sulfuric acid) was mixed with 700 μL of sulfuric acid (pH 3.0) and the mixture was vortexed for 30 s. Then, 100 μL of 10 mM IBP in phosphate buffer pH 7.2 was added and vortexed. The total volume was set to 1 mL by adding 100 μL of 10 mM hydrogen peroxide. The components were mixed in the respective order and the reaction mixtures were placed in a 37 °C water bath. The samples were analyzed after 0, 10, 60, 80, and 120 min of incubation. “Blank” samples did not contain IBP.

At the end of each incubation period, the mixtures were acidified with 20 μL of 2 M sulfuric acid and 50 μL of 10 mM salicylic acid as an internal standard was added (final concentration 0.467 mM). The samples were vortex mixed and extracted twice with 2 mL of diethyl ether. The combined ether extracts were evaporated under N_2_ gas. Before HPLC and LC-MS analysis, the dry residue was reconstructed in 100 μL of acetonitrile.

### 4.3. Udenfriend’s Assay

The assay was performed as reported earlier [38]. To a test tube, 3.0 mL of distilled water, 4.0 mL of 2.5 mM IBP solution in 0.1 M phosphate buffer (pH 7.2), 1.0 mL of 10 mM ascorbic acid, 1.0 mL of 2.4 mM Na_2_EDTA, and 1.0 mL of 2.0 mM Fe(NH_4_)_2_(SO_4_)_2_ solution were added, in the order of listing. The mixture was vortexed after adding each component. Then, it was incubated in a water bath at 37 °C for 2 h with gentle mechanical shaking, and 1.0 mL aliquot was taken from the mixture at 0, 10, 60, 80, and 120 min.

To the 1.0 mL aliquot, 1.0 mL of 0.4 M ice-cold perchloric acid and 100 μL of 10 mM of salicylic acid (as an internal standard) were added (final concentration of 0.476 mM). The acidic solution was cooled in icy water and extracted twice with 3.0 mL of diethyl ether. The combined ether layers were evaporated under N_2_ gas. Before analysis, the dry residue was reconstructed in 100 μL of acetonitrile.

### 4.4. Animals and Experimental Procedure

The experiments were performed following the protocol before [14,15,16]. Male Wistar rats (9–11 weeks old, weighing 250–300 g; TOXI-COOP, Hungary, Budapest) were separated into two groups: Group I (control) and Group II (diabetic) animals (*n* = 5 per group). Experimental diabetes was induced by a 65 mg/kg bw intravenous injection of streptozotocin (STZ) one week before the intestinal perfusion. Blood glucose levels were tested before the STZ-treatment and before starting the experiments. The experimental animals were provided standard chaw and water ad libitum.

The animals had fasted for 18–20 h before the experiments, then anesthetized with an intraperitoneal injection of urethane (1.2 g/kg bw). The abdomen was opened by a midline incision. A jejunal loop (length of the jejunal loop about 10 cm) was “in vivo” isolated and cannulated at its proximal and distal ends. Body temperature was maintained at 37 °C using a heat lamp.

Perfusion through the lumen of the jejunal loop with an isotonic medium containing 250 µM ibuprofen was carried out at a rate of 13 mL/min in a recirculation mode. Perfusate samples (250 μL) were collected at selected timepoints (15, 30, 45, 60, 75, and 90 min) from the perfusion medium flowing out from the intestinal loop. The initial volume of the perfusate was 15 mL and its temperature was maintained at 37 °C. 

For parallel investigation of the biliary excretion, the bile duct was cannulated with PE-10 tubing. The bile outflow was collected in 15-min periods into tared Eppendorf tubes placed in ice. The collected samples were stored in a deep freezer (−70 °C) until analysis. Bile flow was measured gravimetrically, assuming a specific gravity of 1.0 [55]. Biliary excretion was expressed as the product of the HPLC peak areas (relative to the internal standard) and the 15-min periods of bile flows (μL/kg/min). The values (arbitrary units) represent the mean ± S.E. of five independent experiments.

### 4.5. Sample Preparation

To 0.1 mL of perfusate sample or 50 μL of bile sample, 20 μL of 2 M sulfuric acid and 10 μL of 10 mM salicylic acid (as an internal standard) were added. (The final concentration of SA in the perfusate and the bile samples was 0.77 mM and 1.25 mM, respectively.) Then, the samples were vortex mixed and extracted twice with 0.5 mL of diethyl ether. After vortexing (30 s) and centrifugation (5 min, 5000 rpm), the ether layers were separated and the combined ether extracts were evaporated under N_2_ gas. Before HPLC-UV and HPLC-MS analysis, the evaporation residue was reconstructed in 100 μL of acetonitrile.

### 4.6. Sample Analysis

#### 4.6.1. HPLC-UV

HPLC-UV analyses (Method I) of the Fenton and the Udenfriend samples were performed on an integrated Agilent 1100 HPLC system equipped with a quaternary HPLC pump, a degasser, an autosampler, a thermostated column compartment, and a diode-array detector. Data were recorded and evaluated by Agilent ChemStation software (Rev.B.03.02-SR2). HPLC-UV analysis (Method II) of the perfusate and bile extracts was performed on an integrated Jasco HPLC (LC-4000) system equipped with a PDA detector and a quaternary HPLC pump, a degasser, an autosampler, a thermostated column compartment, and a PDA detector. Data were recorded and evaluated by ChromNAV Data System (Ver.2). 

Method I. Separation of compounds was performed on a Teknokroma (NUCLEOSIL C18) (4.6 mm × 250 mm, 5 µm particle size) column with a Teknokroma (ODS cartridge, 1 cm × 0.32 cm) guard column at 40 °C. The mobile phase consisted of 0.02 M phosphate buffer pH 2.5 (A) and acetonitrile (B) with a flow rate of 1.5 mL/min. The analyses (detection at 220 nm) were performed using the following gradient profile: 20% B for 15 min followed by a 5 min linear gradient to 60% B, a 4 min isocratic elution followed by a 2 min linear gradient to 20% B, and a 4 min equilibration. The injected volume was 10 µL.

Method II. Separation of compounds was performed on a Zorbax SB C-18 (4.6 mm × 150 mm, 5µm particle size) column with a Teknokroma (ODS cartridge, 1 cm × 0.32 cm) guard column at 40 °C. The mobile phase consisted of 0.02 M phosphoric acid pH 2.5 (A) and acetonitrile (B) with a flow rate of 1.6 mL/min. The analyses (detection at 220 nm) were performed using the following gradient profile: 20% B for 5 min followed by a 0.5 min linear gradient to 30% B, a 4.5 min isocratic elution followed by a 0.1 min linear gradient to 60% B, a 4.9 min isocratic elution followed by a 0.1 min linear gradient to 20% B, and a 4.9 min equilibration. The injected volume was 10 µL.

#### 4.6.2. HPLC-HESI-MS

To identify the metabolites, a Thermo Dionex UltiMate 3000 liquid chromatograph (Dionex, Sunnyvale, CA, USA) connected to a Thermo Q Exactive Focus quadrupole-Orbitrap hybrid mass spectrometer (Thermo Fisher Scientific, Waltham, MA, USA) was used. Data acquisition and analysis were performed using the Q Exactive Focus 2.1, Xcalibur 4.2., and FreeStyle 1.8 software (Thermo Fisher Scientific, Waltham, MA, USA).

The HPLC separation was performed on an XTerra MS C18 column (150 mm × 2.1 mm, 3.5 µm) with XTerra MS C18 precolumn (5 mm × 2.1 mm, 3.5 µm) at 40 °C. The injection volume was 5 µL and the flow rate was 0.4 mL/min. The tray of the autosampler vials was thermostated at 25 °C. A binary gradient of the eluents is as follows.

Eluent: 5 mM ammonium-acetate/5 mM acetic acid in water (A) and methanol (B). Gradient: 10% B for 1 min, followed by a 9 min linear gradient to 90% B, a 2 min isocratic elution followed by a 0.5 min linear gradient to 10% B, and a 2.5 min equilibration.

Analysis of the compounds was performed in HESI negative ionization modes with the following parameters: spray voltage 3.0 kV; probe heater temperature 300 °C; capillary temperature 350 °C; spray and auxiliary N_2_ gas flows 20 and 5 arbitrary units, respectively; S-Lens RF level 50%; automatic gain control 1 × 10^6^; resolution (at 200 *m*/*z*) 70,000; data acquisition range *m*/*z* 100–1000, in full scan mode.

### 4.7. Statistical Analysis

HPLC-UV integrated peak areas (relative to the internal standard) of oxidized ibuprofen metabolites were qualitatively analyzed in the Fenton and Udenfriend experiments based on incubation time. Biliary excretion was expressed as the product of the HPLC peak areas (relative to the internal standard) and the 15-min period of bile flows (μL/kg/min). The values (arbitrary units) represent the mean ± S.E. of five independent experiments. The difference among groups was determined by an SPSS independent t-test. Significant differences from the control value: * *p*< 0.05 and ** *p* < 0.01.

### 4.8. Ethical Approval

The study was designed and conducted according to European legislation (Directive 2010/63/E.U.) [56] and Hungarian Government regulation (40/2013, II. 14.) [57] on the protection of animals used for scientific purposes. The project was approved by the Animal Welfare Committee of the University of Pécs and by the Government Office of Baranya County (license No. BAI35/51-61/2016 and license supplement (supplement No. BAI35/90-5/2019)).

## 5. Conclusions

The present results show that one of the main hydroxylated IBP derivatives formed in the non-enzyme-catalyzed oxidation reactions (Fenton and Udenfriend tests) is a hydroxylated IBP derivative (**8**). Since the formation of neither 2′-OH-IBP nor OH(Ar)-IBP (**8)**—the possible structures of the product—has not been reported in CYP-catalyzed reactions, formation of this derivative might be used as a biomarker of oxidative stress in living organisms.

Contrary to 4-nitrophenol [16] and capsaicin [58]—both are phenolic derivatives—the glucuronide conjugate of ibuprofen could be detected only in a trace amount in the small intestinal perfusates. On the other hand, IBP (**1**), IBP-GLU (**6**), and IBP-TAU (**7**) were excreted by the liver. However, no specific non-enzymatic hydroxylation product could be detected.

The results are in agreement with the previous experimental findings demonstrating decreased expression of the organic anion transporters P-gp (MDR1), MRP2, and BCRP in the liver of the STZ-treated (hyperglycemic) animals. Such changes impact the pharmacokinetics of drugs administered in hyperglycemic individuals.

## Data Availability

Primary research data are stored in the Institute of Pharmaceutical Chemistry, University of Pécs, Pécs, Hungary.

## Supplementary Materials

The following supporting information can be downloaded at: https://www.mdpi.com/article/10.3390/molecules27134000/s1.

## Sample Availability

Samples of the compounds are not available from the authors.
