# Supplementary material for: The Intestinal and Biliary Metabolites of Ibuprofen in the Rat with Experimental Hyperglycemia
Source: Molecules. 2022 Jun 22;27(13):4000. doi: 10.3390/molecules27134000 (PMC9268267; doi:10.3390/molecules27134000)

molecules

HPLC studies on the effect of experimental hyperglycemia on intestinal and biliary metabolites of ibuprofen in the rat. Lack of non-enzyme-catalyzed oxidative metabolites.

Supplementary Materials

Hawsar Othman Mohammed [1,2], Attila Almasi[1], Szilard Molnar[1] and Pal Perjesi[1*]

[1]Institute of Pharmaceutical Chemistry, Faculty of Pharmacy, University of Pécs, Pécs, Hungary
[2]College of Veterinary Medicine, University of Sulaimani, Sulaymaniyah, Kurdistan Region, Iraq

***Corresponding Author**
Pál Perjési, PhD
Institute of Pharmaceutical Chemistry
University of Pécs
E-mail: pal.prejesi@gytk.pte.hu

Figure S1. HPLC-UV chromatogram (Method I) of the blank Fenton extract. Salicylic acid (internal standard) t_R=7.74 min.

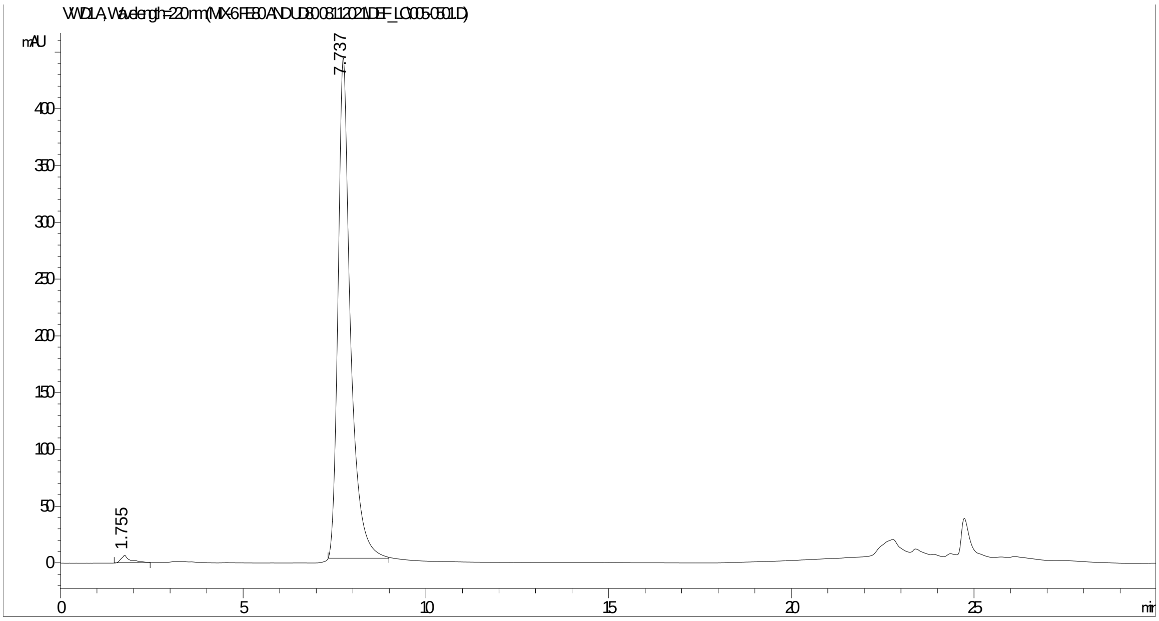

Figure S2. HPLC-UV chromatogram (Method I) of the Fenton extract of IBP (80-minute). Salicylic acid (internal standard) t_R=7.69 min, 2-hydroxyibuprofen (**3**) t_R=14.94 min, 1-hydroxyibuprofen (**2**) t_R=19.92 min, dihydroxyibuprofen (**10**) t_R=21.09 min, aromatic-hydroxylated ibuprofen (**8**) t_R=22.05 min, ibuprofen t_R=23.68 min.

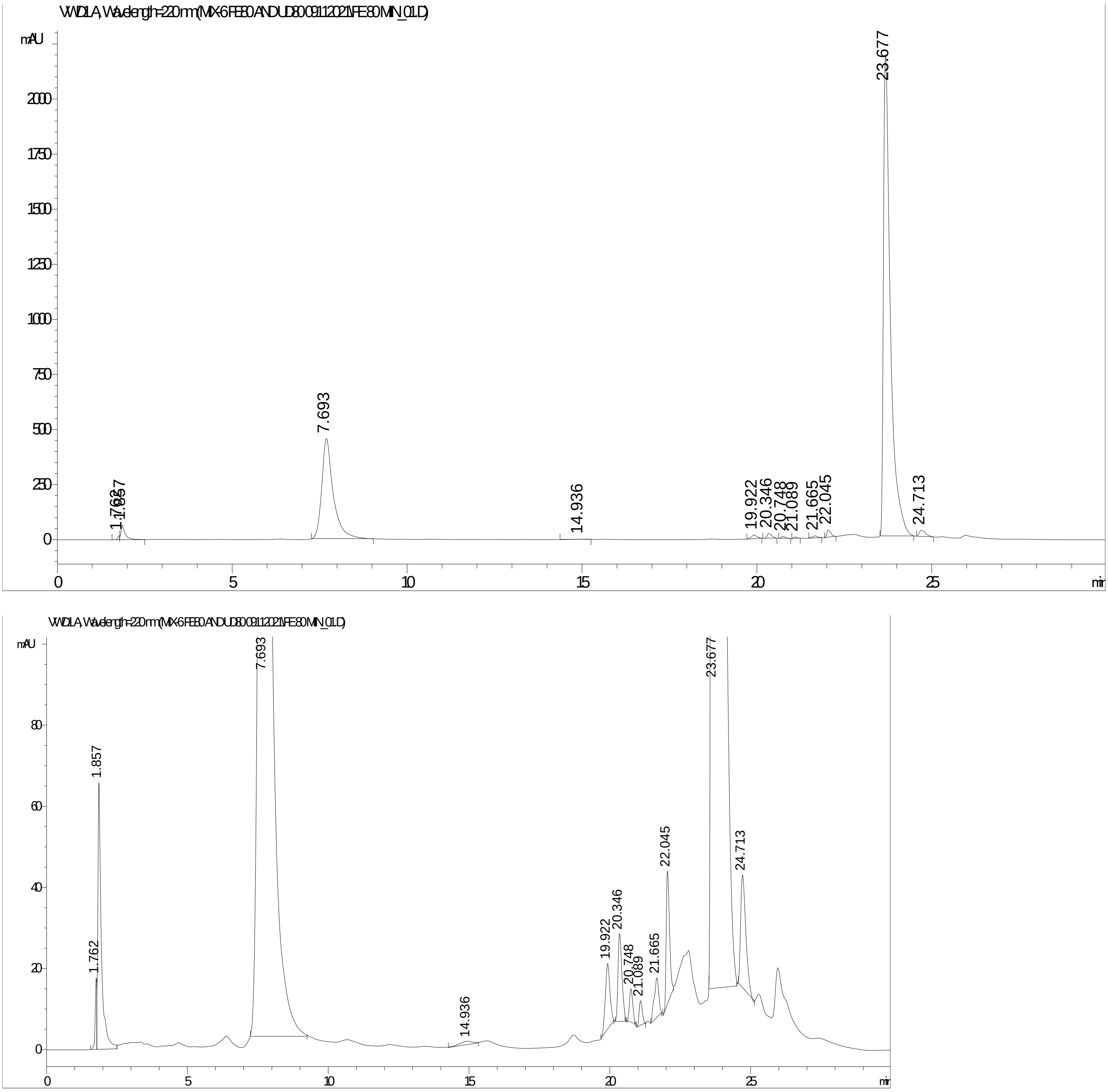

Figure S3. HPLC-MS spectrum of the 1-hydroxyibuprofen (**2**) (1-OH-IBP) standard. ($C_{13}H_{17}O_3$; Exact mass: 221.1178) Mass error: -0.90 ppm. (The m/z 204.9982 signal is a background ion.). The following fragments are formed in the ionization source: 443.2436: $[2M-H]^-$, 221.1176: $[M-H]^-$, 204.9982: unknown; 177.1274: $[M-H-CO_2]^-$, 159.1168: $[M-H-CO_2-H_2O]^-$.

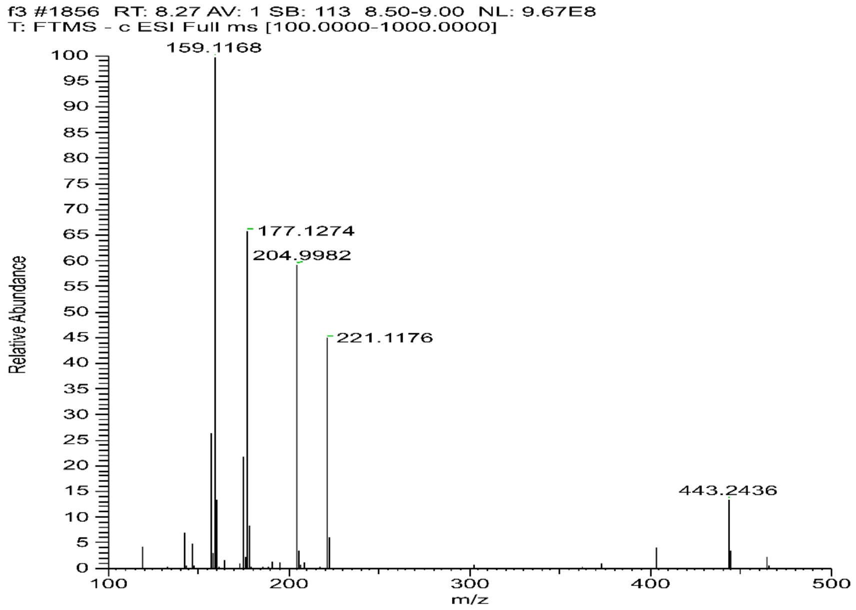

Figure S4. HPLC-MS spectrum of the 2-hydroxyibuprofen (**3**) (2-OH-IBP) standard. ($C_{13}H_{17}O_3$; Exact mass: 221.1178) Mass error: -0.45 ppm. (The m/z 204.9982 signal is a background ion.) The following fragments are formed in the ionization source: 443.2437: $[2M-H]^-$, 221.1177: $[M-H]^-$, 204.9982: unknown, 133.0645: unknown.

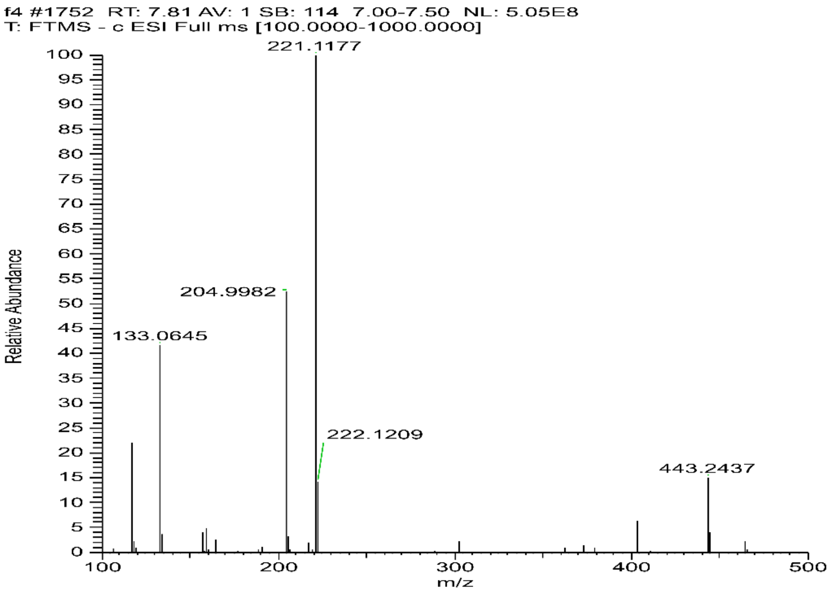

Figure S5. HPLC-MS spectrum of the carboxyibuprofen (**5**) (HOOC-IBP) standard. ($C_{13}H_{15}O_4$; Exact mass: 235.0970) Mass error: -0.43 ppm. The m/z 204.9982 signal is a background ion.) The following fragments are formed in the ionization chamber: 471.2020: $[2M-H]^-$, 235.0969: $[M-H]^-$, 204.9982: unknown, 191.1068: $[M-H-CO_2]^-$.

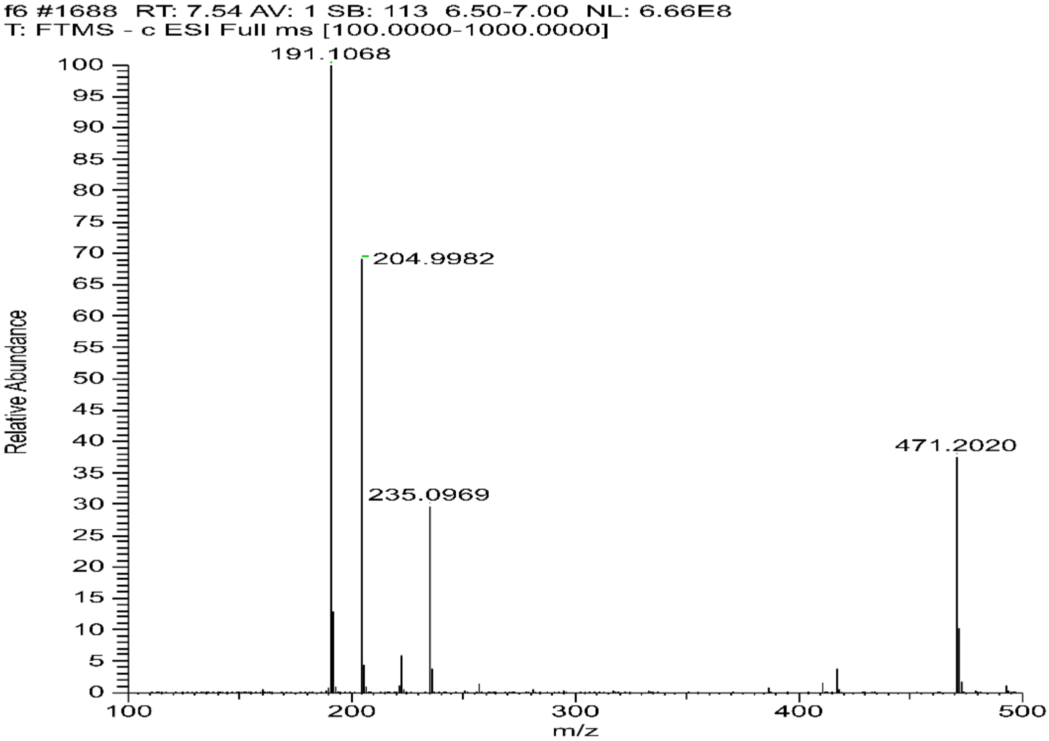

Note: In the mass spectra of standards **2** (Figure S3), **3** (Figure S4), and **5** (Figure S5), the peak of m/z 204.9982 systematically appears without collision of the compounds. The exact mass of ibuprofen (in negative mode) is 205.1229. The m/z 204.9982 amu is 608 ppm smaller. Since MS measurements were performed with an instrument with high mass accuracy, the m/z 204.9982 peak was defined as unknown.

Figure S6. A HPLC-MS spectrum of the hydroxylated ibuprofen (**8**) (X-OH-IBP) derivative. ($C_{13}H_{17}O_3$; Exact mass: 221.1178) Mass error: -1.36 ppm. The following fragments are formed in the ionization source:443.2433: $[2M-H]^-$, 221.1175: $[M-H]^-$, 177.1274: $[M-H-CO_2]^-$. **B.** HPLC-MS fragmentation of the hydroxylated ibuprofen (**8**) (X-OH-IBP) derivative. ($C_{13}H_{17}O_3$; Exact mass: 221.1178)

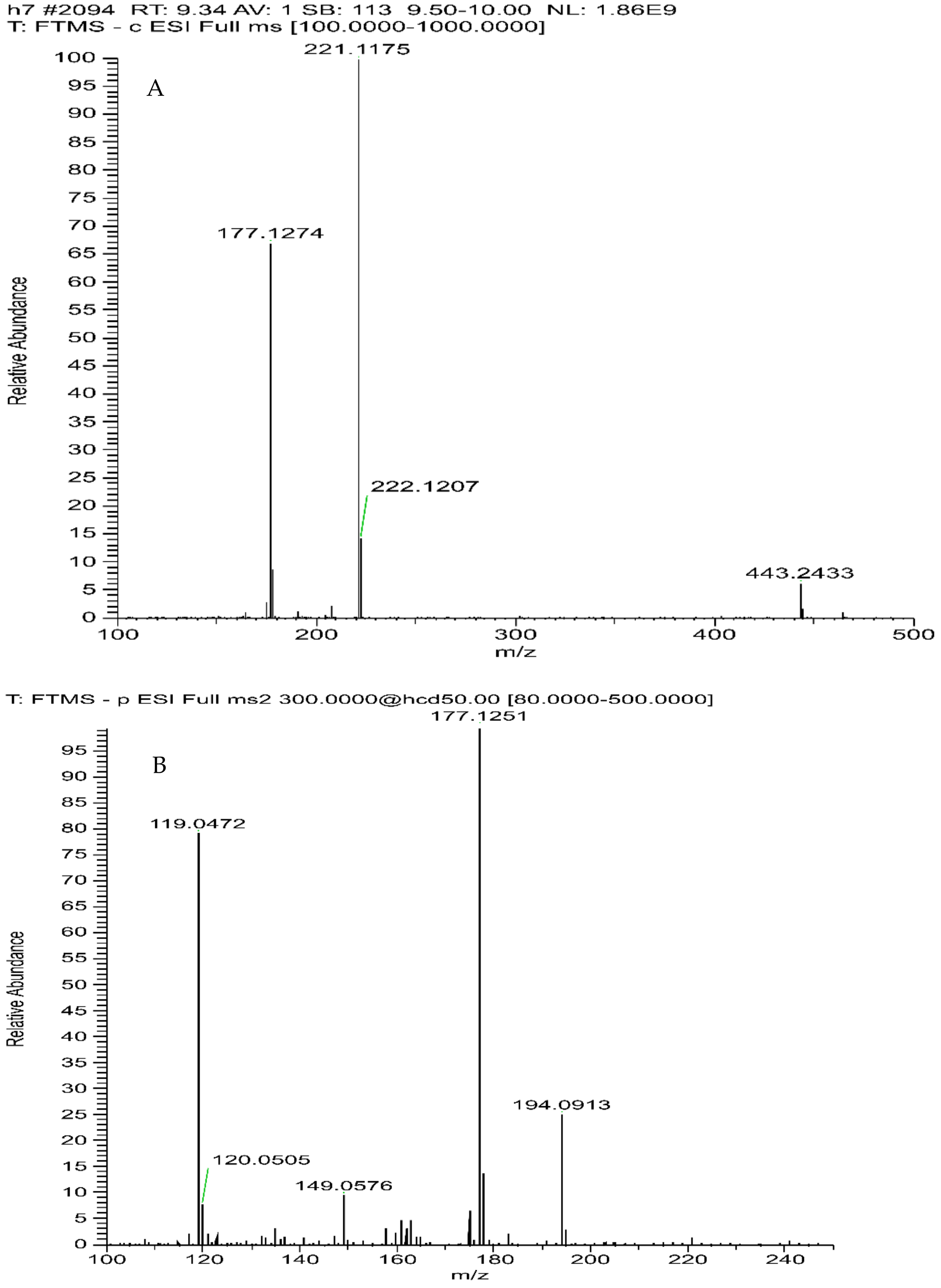

Figure S7. HPLC-MS spectrum of the dihydroxyibuprofen (**10**) (OH-IBP-OH) derivative. ($C_{13}H_{17}O_4$; Exact mass: 237.1127) Mass error: -0.84 ppm. The following fragments are formed in the ionization source: 237.1125: $[M-H]^-$, 193.1224: $[M-H-CO_2]^-$.

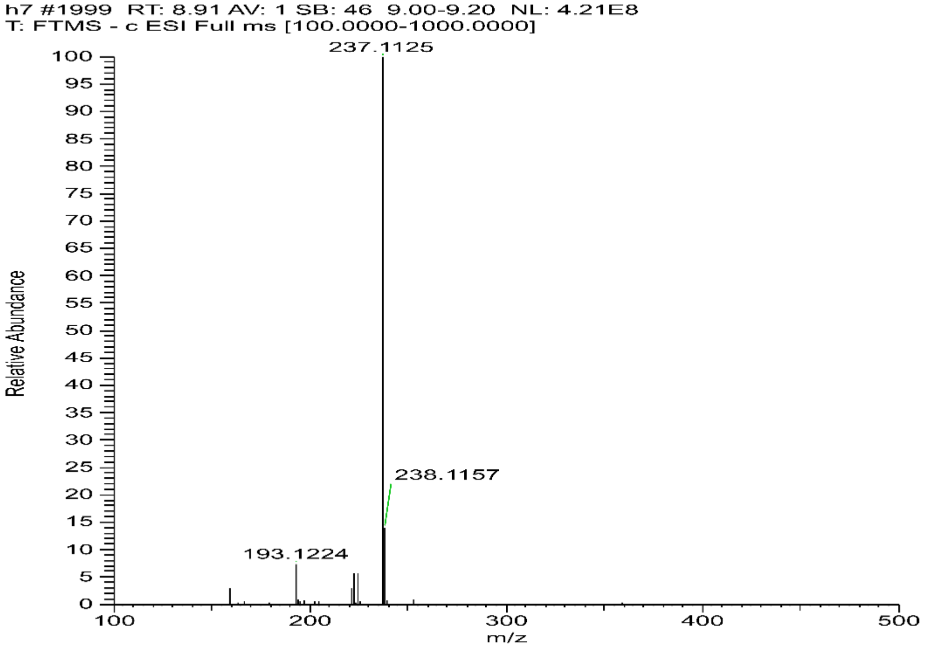

Figure S8. Change in the HPLC-UV integrated peak areas (relative to the internal standard) of the 2-OH-IBP (**3**), 1-OH-IBP (**2**), OH-IBP-OH (**10**), and the X-OH-IBP (**8**) derivatives ibuprofen(IBP)-metabolites in the diethyl ether extracts of Fenton incubation mixtures (Method I).

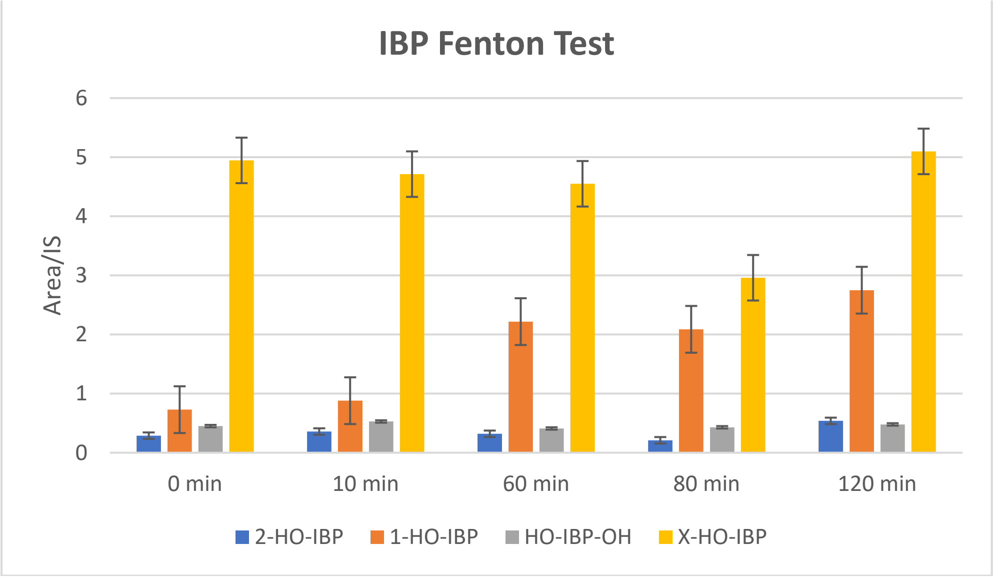

Figure S9. HPLC-UV-Vis chromatogram (Method I) of the blank Udenfriend extract. Salicylic acid (internal standard.) t_R=7.74 min.

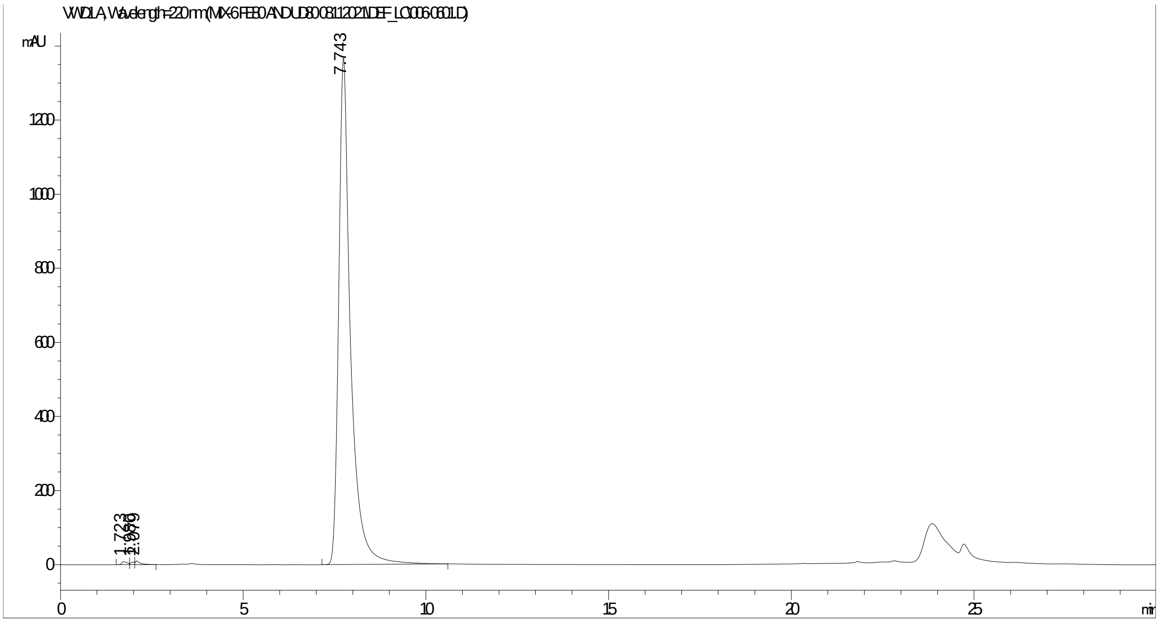

Figure S10. HPLC-UV chromatogram (Method I) of the Udenfriend extract of IBP (80-minute). Salicylic acid (internal standard) t_R=7.71 min, 2-hydroxyibuprofen (**3**) t_R=14.92 min, dihydroxyibuprofen (**10**) t_R=21.07 min, aromatic-hydroxylated ibuprofen (**8**) t_R=22.03 min, ibuprofen t_R=23.68 min.

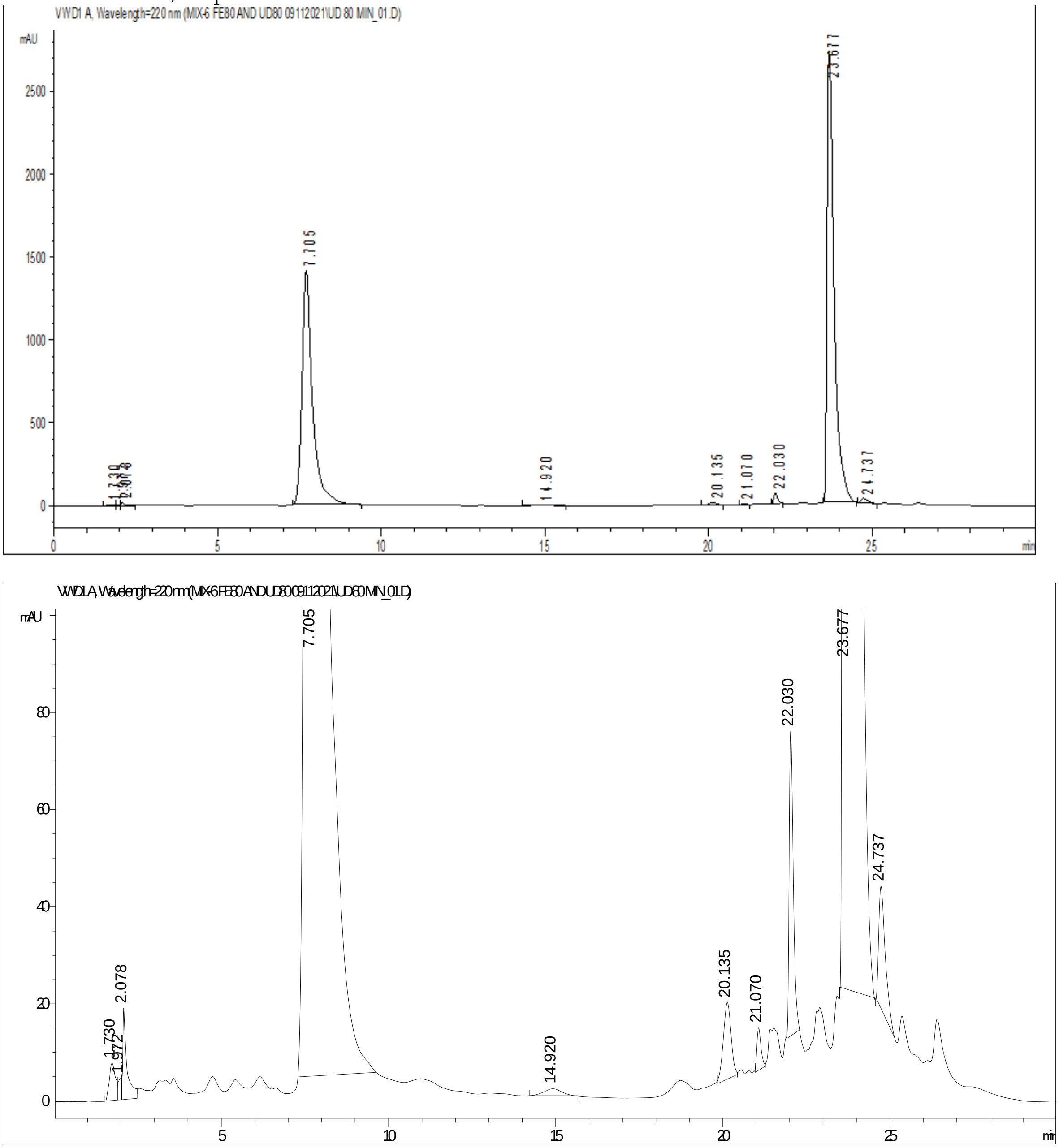

Figure S11. Change in the HPLC-UV integrated peak areas (relative to the internal standard) of the 2-OH-IBP (**3**), OH-IBP-OH (**10**), and the X-OH-IBP (**8**) derivatives in the diethyl ether extracts of the Udenfriend's incubation mixtures (Method I).

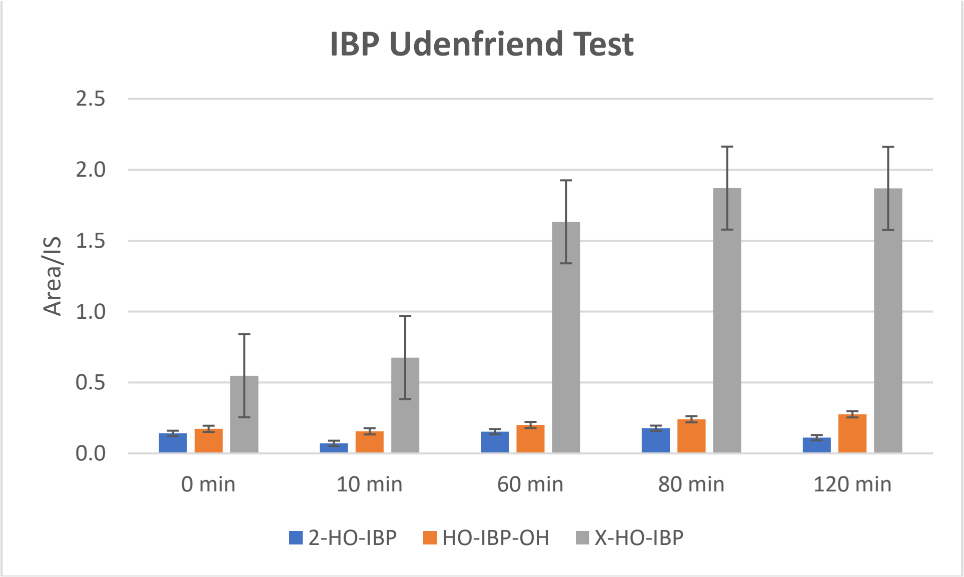

Figure S12. HPLC-UV chromatogram (Method II) of the main oxidative metabolite and the IBP-GLU standards of IBP in ACN (30 μg mL^{-1} each). The retention times of the separated standards as follows: **1**. 3-hydroxyibuprofen (t_R=1.50 min), **2**. salicylic acid (internal standard) (t_R=4.92 min), **3.** 2-hydroxyibuprofen (t_R=7.85 min), **4**. carboxyibuprofen (t_R=8.45 min), **5**. 1-hydroxyibuprofen (t_R=9.63 min), **6**. ibuprofen- -D-glucuronide (t_R=11.87 min), **7**. ibuprofen (t_R=13.67 min). (The negative peak in the chromatogram is due to the quick change of mobile phase (solvent B) in the gradient profile.)

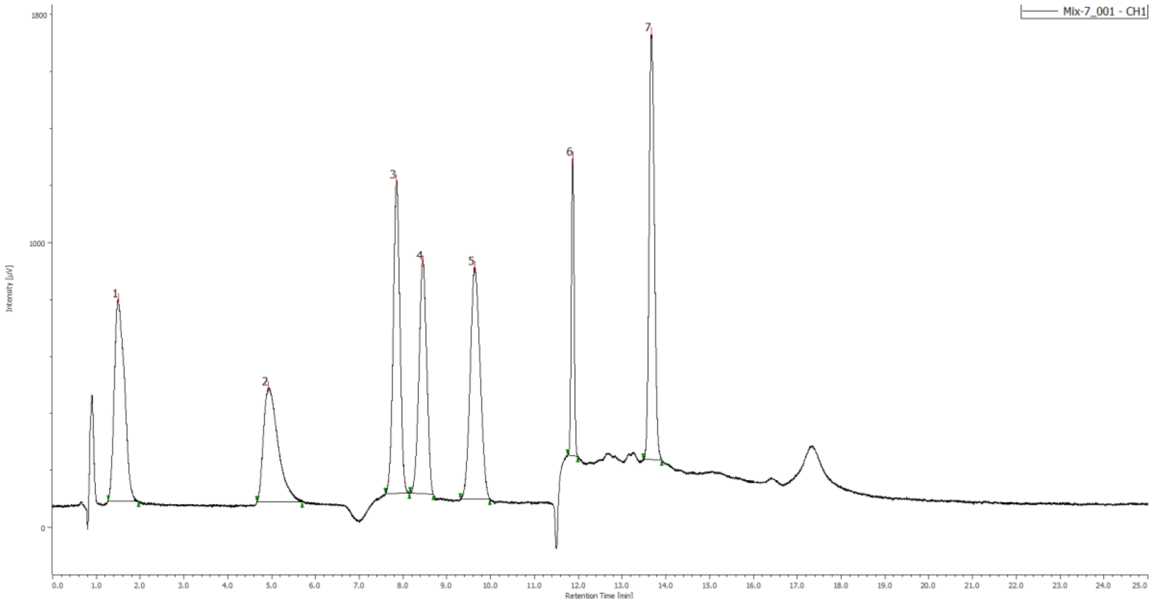

Figure S13. HPLC-UV chromatogram (Method II) of the intestinal perfusate extract of the control rats. (The negative peak in the chromatogram is due to the quick change of mobile phase (solvent B) in the gradient profile.)

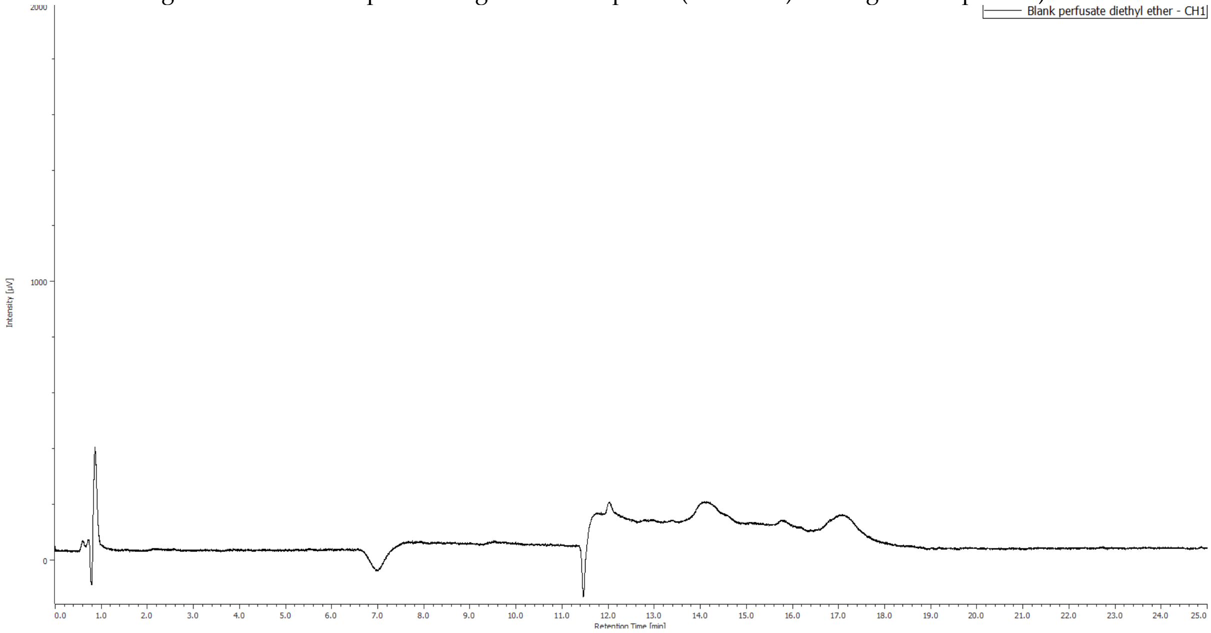

Figure S14. **A**. HPLC-UV chromatogram (Method II) of the intestinal perfusate extract of the control rats (0 minute) (**1**) Salicylic acid (internal standard) t_R=4.35 min, (**2**) ibuprofen t_R. 13.40 min. **B**. HPLC-UV chromatogram of the intestinal perfusate extract of the STZ-treated rats (0 minute). (**1**) salicylic acid (internal standard) t_R=4.50 min, (**2**) ibuprofen t_R=13.40 min. (The negative peak in the chromatogram is due to the quick change of mobile phase (solvent B) in the gradient profile.)

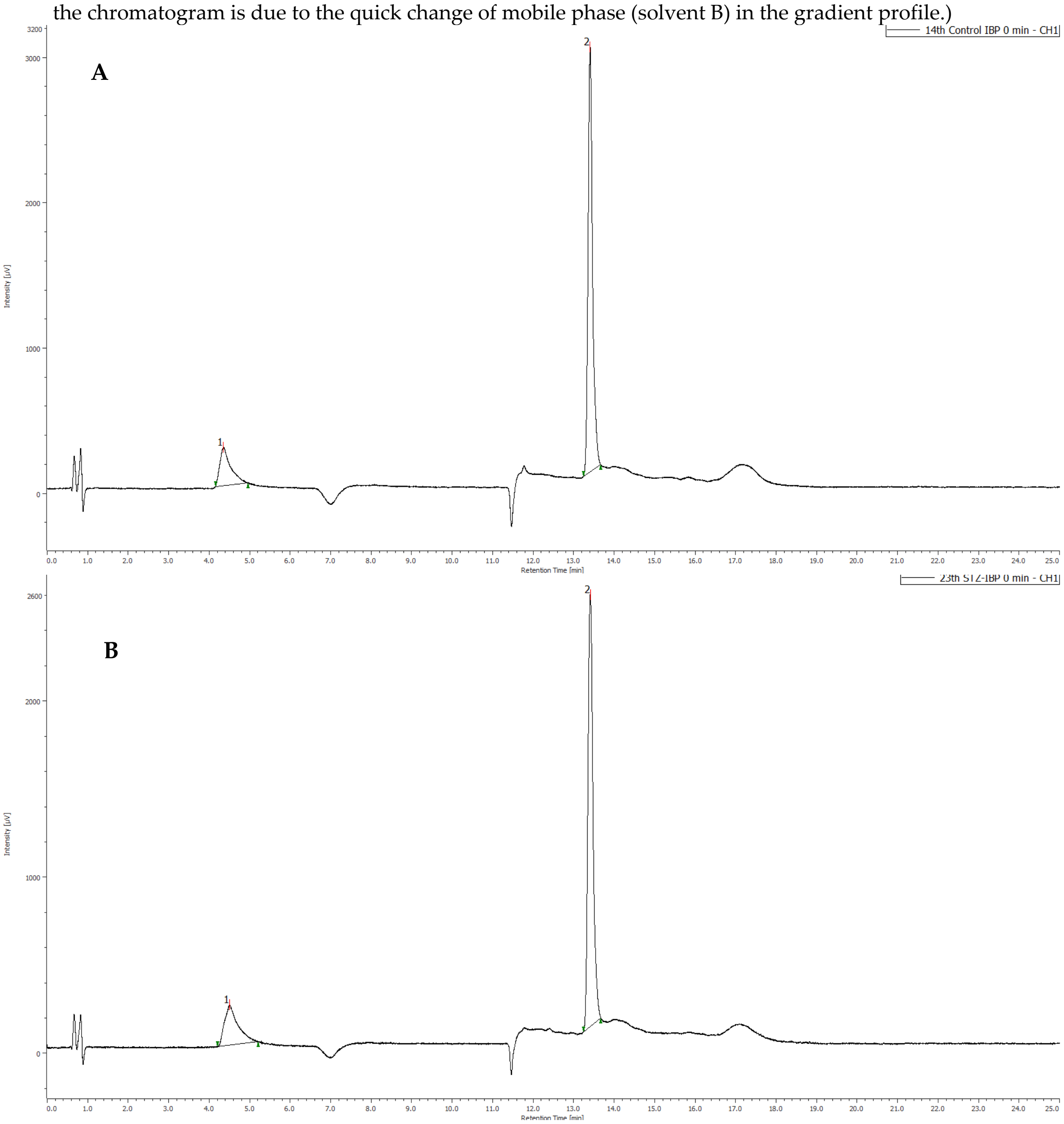

Figure S15. **A**. HPLC-UV chromatogram (Method II) of the intestinal perfusate extract of the control rats (90-minute). (**1**) Salicylic acid (internal standard) t_R=4.34 min, (**2**) ibuprofen t_R=13.39 min. **B**. HPLC-UV chromatogram (Method II) of the intestinal perfusate extract of the STZ-treated rats (90-minute). (**1**) salicylic acid (IS) t_R. 4.46 min, (**2**) ibuprofen t_R=13.38 min. (The negative peak in the chromatogram is due to the quick change of mobile phase (solvent B) in the gradient profile.)

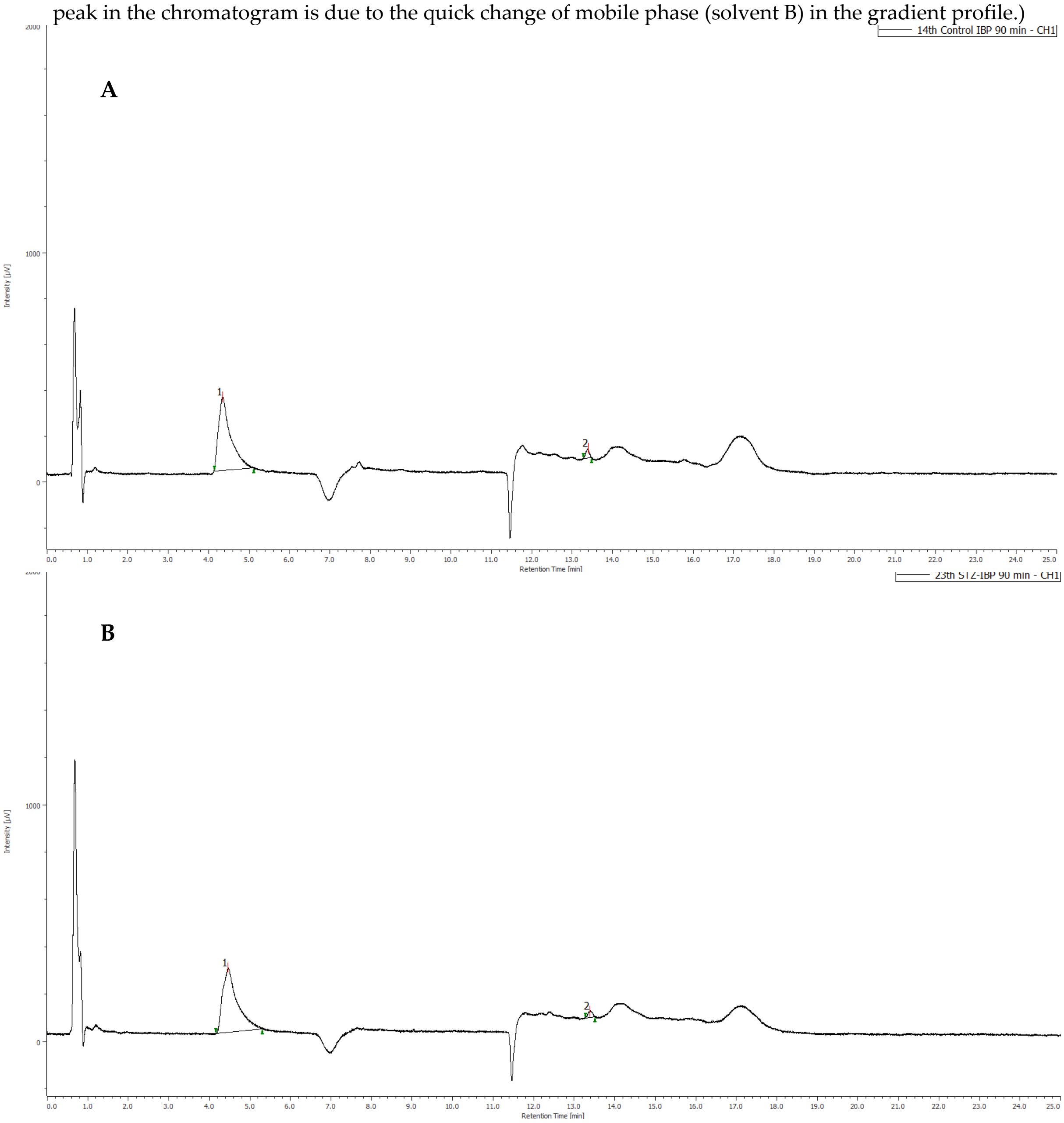

Figure S16. HPLC-MS spectrum of the ibuprofen- -D-glucuronide (**6**) (IBP-GLU)) standard. ($C_{19}H_{25}O_8$; Exact mass: 381.1549). Mass error = 0.79 ppm. The following fragments are formed in the ionization source: 763.3177: $[2M-H]^-$, 204.9982: unknown.

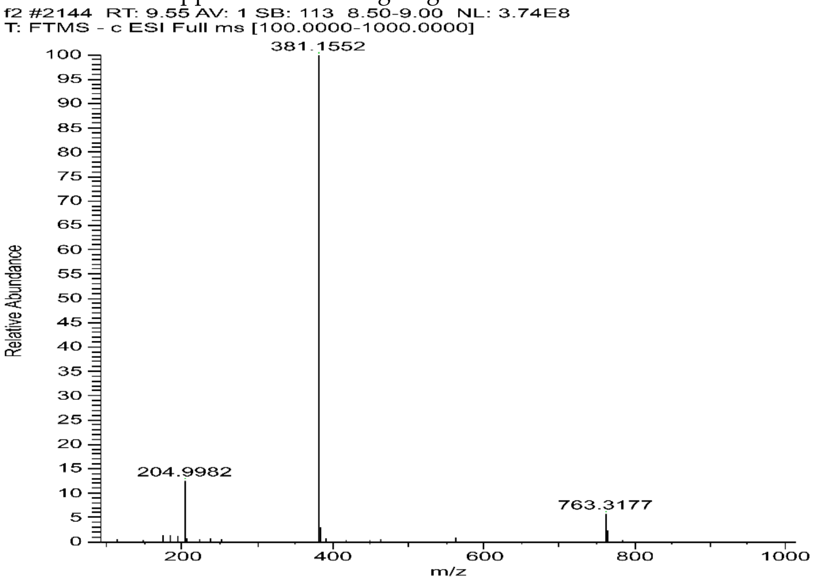

Figure S17. HPLC-UV chromatogram (Method II) of the bile extract of the control rats.

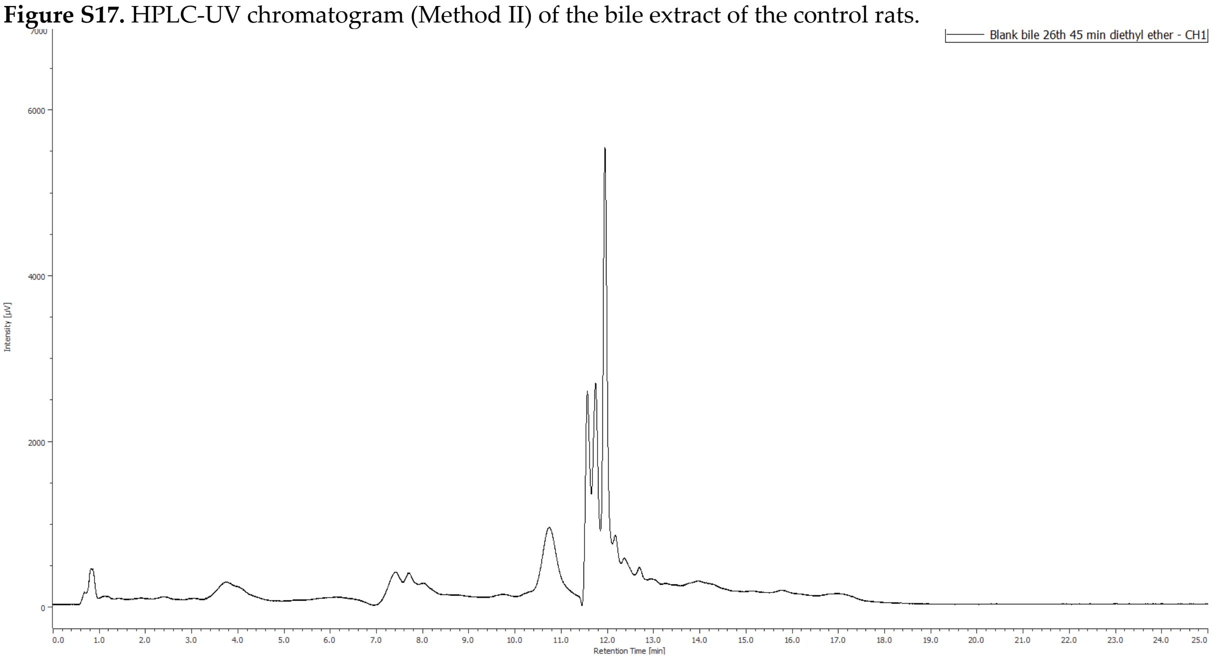

Figure S18. A. HPLC-UV chromatogram (Method II) of the bile extract of the control rats (15 min). (**1**) Salicylic acid (internal standard) t_R=4.52 min, (**2**) 2-hydroxyibuprofen t_R=7.68 min, (**3**) ibuprofen-glucuronide t_R=11.78 min, (**4**) ibuprofen t_R=13.41 min. **B**. HPLC-UV chromatogram (Method II) of the bile extract of the STZ-treated rats (15 min). (**1**) Salicylic acid (IS) t_R=4.45 min, (**2**) 2-hydroxyibuprofen t_R=7.45 min, (**3**) ibuprofen-glucuronide t_R=11.77 min, (**4**) ibuprofen t_R=13.36 min.

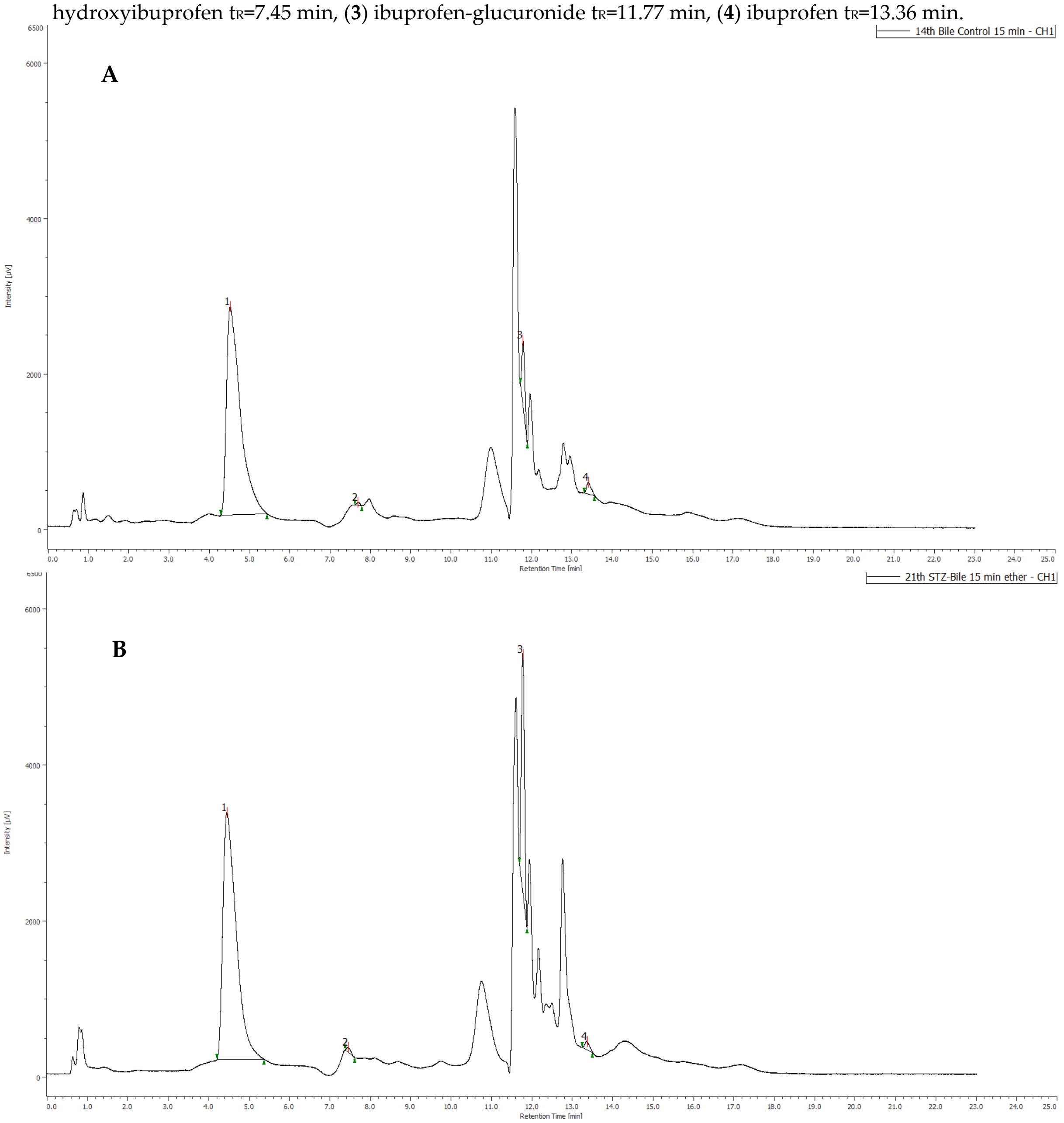

Figure S19. A HPLC-UV chromatogram (Method II) of the bile extract of the control rats (90 min).; (**1**) Salicylic acid (internal standard) t_R=4.62 min, (**2**) 2-hydroxyibuprofen t_R=7.61 min, (**3**) ibuprofen-glucuronide t_R=11.79 min, (**4**) ibuprofen t_R=13.39 min. **B**. HPLC-UV chromatogram (Method II) of the bile extract of the STZ-treated rats (90 min). (**1**) Salicylic acid (IS) t_R=4.48 min, (**2**) 2-hydroxyibuprofen t_R=7.51 min, (**3**) ibuprofen-glucuronide t_R=11.78 min, (**4**) ibuprofen t_R=13.37 min.

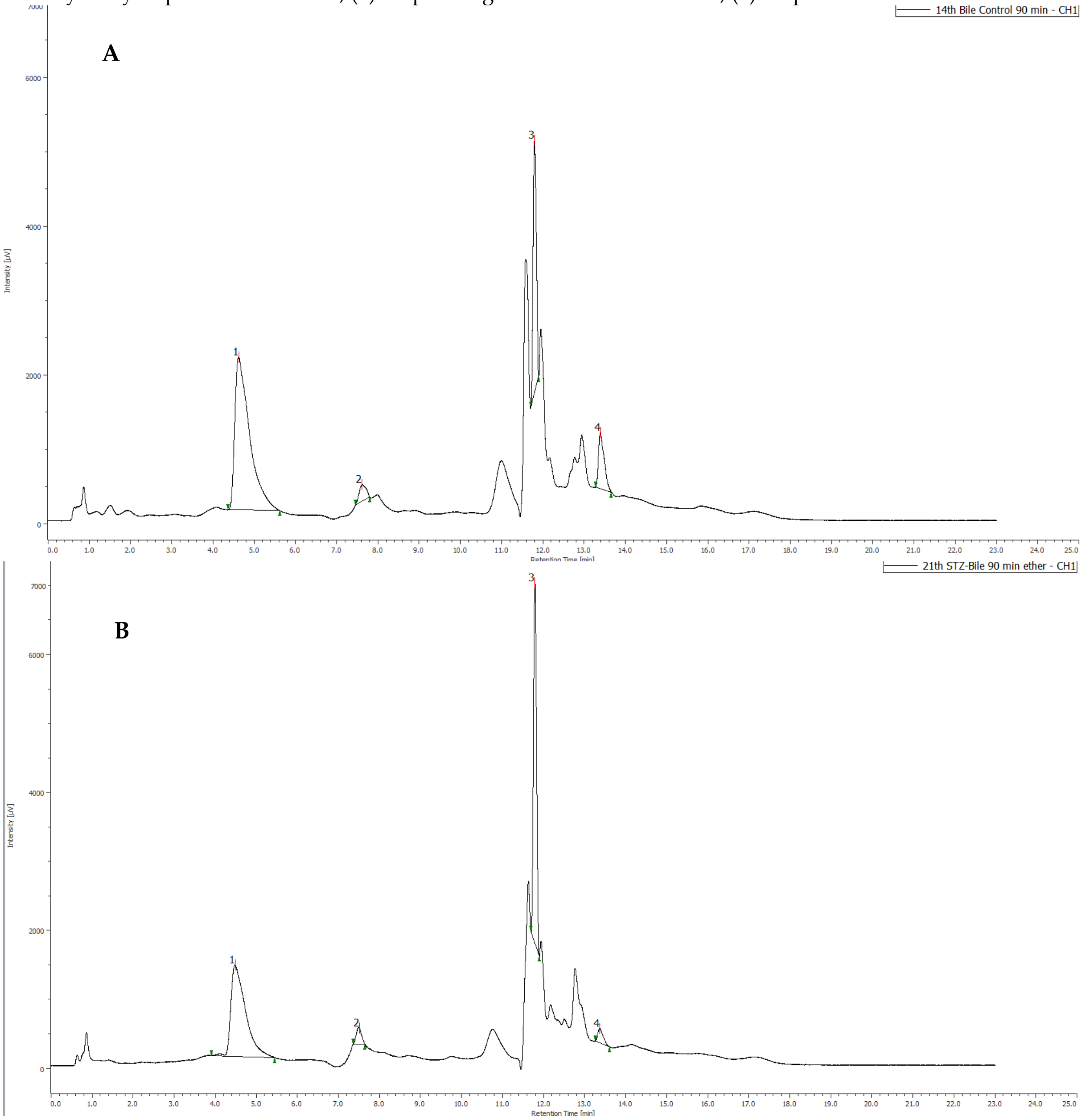

Figure S20. HPLC-MS spectrum of the hydroxyibuprofen-glucuronide (**9**) (X-OH-IBP-GLU) derivative. ($C_{19}H_{25}O_9$; Exact mass: 397.1499) Mass error: 0.50 ppm.

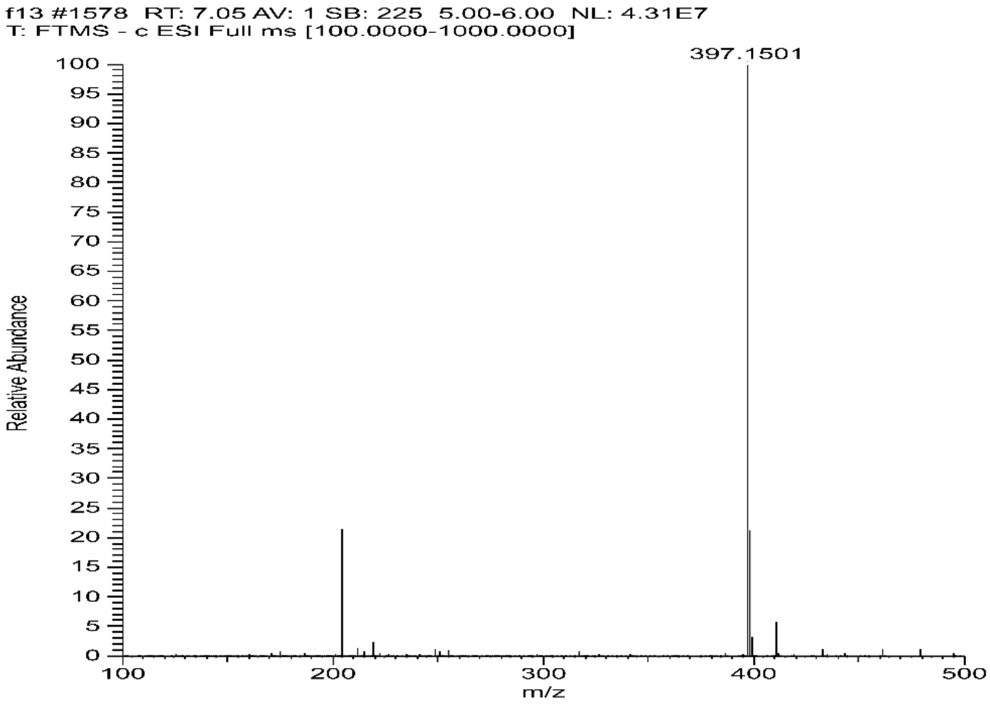

Figure S21. HPLC-MS spectrum of the ibuprofen-taurate (**7**) (IBP-TAU) derivative. ($C_{15}H_{22}NO_4S$; Exact mass: 312.1270). Mass error: 1.60 ppm. The unknown signals are from the background.

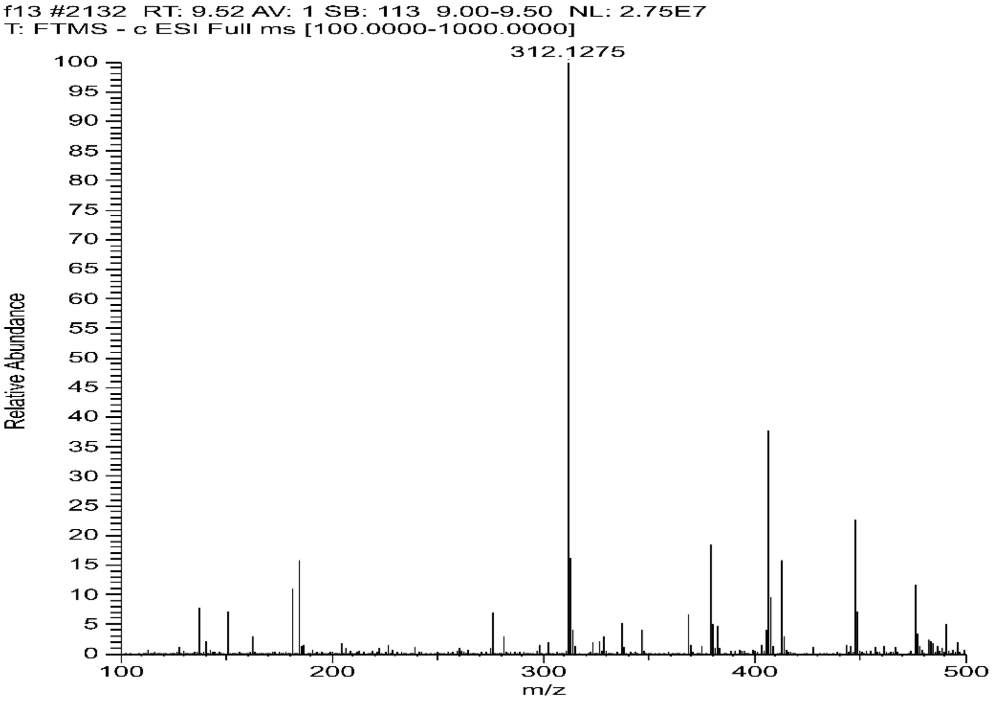

Supplement: Supplementary file 1 [file molecules-27-04000-s001.zip › molecules-1781389-supplementary.pdf]
